# Deficiency in the Treatment Description of mTOR Inhibitor Resistance in Medulloblastoma, a Systematic Review

**DOI:** 10.3390/ijms23010464

**Published:** 2021-12-31

**Authors:** Hajar Alammar, Rayan Nassani, Mana M. Alshehri, Alaa A. Aljohani, Bahauddeen M. Alrfaei

**Affiliations:** 1 College of Medicine, King Saud bin Abdulaziz University for Health Sciences, Ministry of National Guard-Health Affairs, Riyadh 11426, Saudi Arabia; H-rjr@hotmail.com (H.A.); aljohania011@ksau-hs.edu.sa (A.A.A.); 2 King Abdullah International Medical Research Center, Department of Cellular Therapy and Cancer Research, King Saud bin Abdulaziz University for Health Sciences (KSAU-HS), Ministry of National Guard-Health Affairs, Riyadh 11426, Saudi Arabia; rayannasani@gmail.com (R.N.); Alshehrima3@ngha.med.sa (M.M.A.)

**Keywords:** mTOR, medulloblastoma, targeted therapy, resistance

## Abstract

Medulloblastoma is a common fatal pediatric brain tumor. More treatment options are required to prolong survival and decrease disability. mTOR proteins play an essential role in the disease pathogenesis, and are an essential target for therapy. Three generations of mTOR inhibitors have been developed and are clinically used for immunosuppression and chemotherapy for multiple cancers. Only a few mTOR inhibitors have been investigated for the treatment of medulloblastoma and other pediatric tumors. The first-generation mTOR, sirolimus, temsirolimus, and everolimus, went through phase I clinical trials. The second-generation mTOR, AZD8055 and sapanisertib, suppressed medulloblastoma cell growth; however, limited studies have investigated possible resistance pathways. No clinical trials have been found to treat medulloblastoma using third-generation mTOR inhibitors. This systematic review highlights the mechanisms of resistance of mTOR inhibitors in medulloblastoma and includes IDO1, T cells, Mnk2, and eIF4E, as they prolong malignant cell survival. The findings promote the importance of combination therapy in medulloblastoma due to its highly resistant nature.

## 1. Introduction

Brain tumors are the most devastating tumors in Children under 14 years old, demographically. They represent the most common tumors among children [1]. Embryonal tumors represent 12.7% of childhood intracranial tumors, while medulloblastoma represents 64.7% of the embryonal tumors. The peak age for diagnosis is 9 years old or younger [1]. Medulloblastoma is a malignant primitive infratentorial tumor [2]. The WHO divided the classification of medulloblastoma into four subgroups, according to their molecular basis. The groups are Wingless (WNT)-activated, Sonic hedgehog (SHH)-activated, Group 3, and Group 4 [3].

The mechanistic target of rapamycin (mTOR) is one of the major pathways that have been activated during medulloblastoma development. It is a master regulator for signaling pathways that organize organismal development and homeostasis, because of its involvement in protein and lipid synthesis besides controlling the cell cycle and the cellular metabolism. mTOR inhibitors are a class of drugs that suppress the mTOR. In the clinic, they are primarily used as immunosuppressants and for the treatment of multiple cancers [4]. Three generations of mTOR-targeted therapy have been developed to date [4]. Many of these agents are undergoing clinical trials for their antineoplastic effects in various tumors, including medulloblastoma. 

The aggressive nature of the medulloblastoma, genetic variation, and the tendency to develop resistance necessitate an update to the conventional treatment [5,6]. Resistance has been observed against mTOR inhibitor-targeted therapy in medulloblastoma. There is a gap of knowledge relevant to the mechanism of resistance of mTOR inhibition in medulloblastoma. The knowledge advancement should include the significant role in medulloblastoma and its potential treatment. 

## 2. Methods

**Figure 1:**
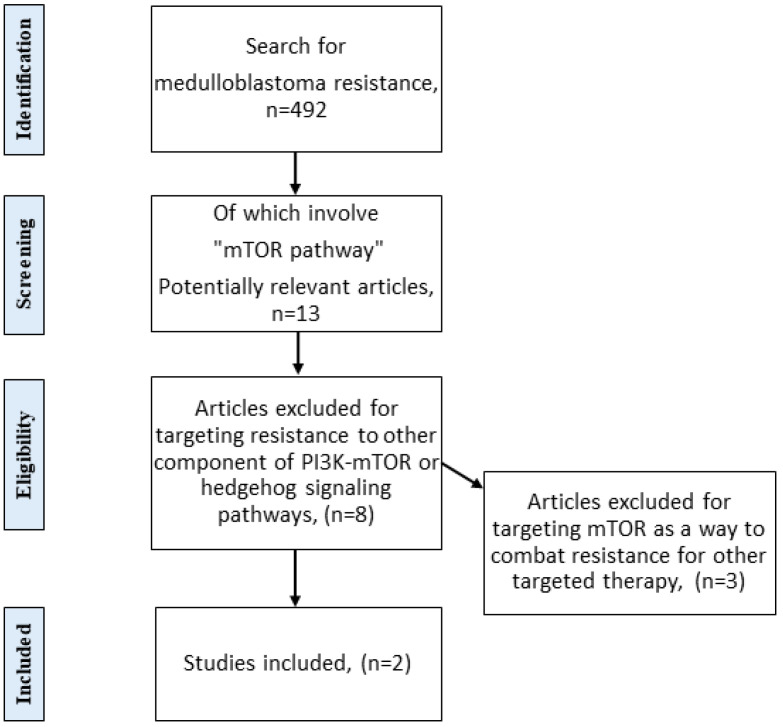
PRISMA flow diagram. The diagram shows preferred reporting items for systematic reviews and meta-analyses. The diagram is a modified version of described PRISMA [7].

A systematic literature review was performed using PubMed and Medline databases and Google Scholar in March 2021. The keywords were “mTOR”, “medulloblastoma”, “treatment”, “drug-resistant”, and “resistance”. The inclusion criteria were studies that mentioned medulloblastoma resistance, specifically in the mTOR pathway. The first search for Medulloblastoma resistance generated 492 articles. The next search focused on mTOR pathways, which reduced the number of articles to 13. The exclusion method excluded 8 articles, and 2 studies were included in the analysis. The preferred reporting items for a systematic review and meta-analyses (PRISMA) were used in this article (Figure 1) [7]. 

## 3. Results and Discussion 

### 3.1. mTOR Molecular Pathway

The mechanistic target of rapamycin (mTOR) is a serin-threonine kinase that belongs to the phosphatidylinositol 3-kinase-related kinases [8]. It compromises part of two distinct protein complexes, mTORC1 and mTORC2 [9]. mTORC1 consists of a regulatory protein associated with mTOR with Raptor, mLST8, PRAS40, and DEPTOR. mTORC2 is characterized by Rictor, the rapamycin-insensitive companion of mTOR, in addition to DEPTOR, mLST8, mSin1, and Protor1/2 [8]. Secondary to external or internal stimuli, both complexes interact in multiple signaling pathways, such as the phosphoinositide 3-kinases (PI3K)/Akt/mTOR signaling cascade and mitogen-activated protein kinase (MAPK) signaling cascade [9]. mTOR has a central role in the regulation of cellular metabolism, including anabolic processes, cellular growth, proliferation, and autophagy [10]. mTOR is also involved in many embryonal processes such as T cell differentiation with neurogenesis, synaptic formation, and neural stem cell regulation [11,12]. The involvement of mTOR in cell differentiation of T helpers: Th1, Th2, and Th17 have been investigated. Inhibition of Rheb (Ras homolog) reduces Th1 differentiation. Similarly, Raptor deletion reduces Th2 differentiation. Raptor deficiency resulted in a significant decrease of Th17^+^ CD27^−^ differentiation to Th17 CD27, which is responsible for producing IFN-ɣ. The deletion of Rictor showed a decrease in both Th1 and Th2 cell differentiation through the downregulation of the AKT and PKC pathways, respectively [11,13].

mTORC1 and mTORC2 expression are important in neural stem cell (NSC) differentiation and prenatal neurogenesis. However, embryonic stem cells express a low level of mTORC1/p70S6K to maintain their undifferentiated state through the upregulation of tuberous sclerosis complex 1/2 (TSC1/2) and DEPTOR, which are mTORC1 inhibitors. The activation of Raptor is specific for NSC differentiation and migration in corticogenesis. In addition, the inhibition of TSC1 and the activation of Rheb promote cortical progenitor cell differentiation and migration. The mTORC1 critical role in maintaining an appropriate cell size and number is compromised when Raptor is deleted resulting in microcephaly. The high expression of mTORC2 is vital for cytoskeletal function and for the interaction with AKT [12]. In cortical neurons, the mTOR signaling is enhanced by dendritic branching when PTEN is deleted. Similarly, the dendritic branching is enhanced by TSC1 deletion and Rheb activation. Although the Dendritic branching is reduced by mTOR inhibition, The reduction is rescued by mTOR resistant mutation [14]. The activation of mTORC1 in the olfactory granule neurons increases dendritic complexity. mTORC1 specific inhibition does not reduce dendrite complexity, but non-specific mTOR inhibition affects dendrite branching [12]. The deletion of TSC1/2 reduces synaptic formation and maintenance [15]. In mice, heterozygous TSC2 develops a dense dendritic spine at the first month of age due to pruning defects. Rapamycin treatment enhances spine elimination in TSC2 heterozygous mice by inducing autophagy. mTORC1 activity is crucial for synapse formation and maintenance. The dysregulation of its activity results in epilepsy and autism spectrum disorder [12]. 

**Figure 2:**
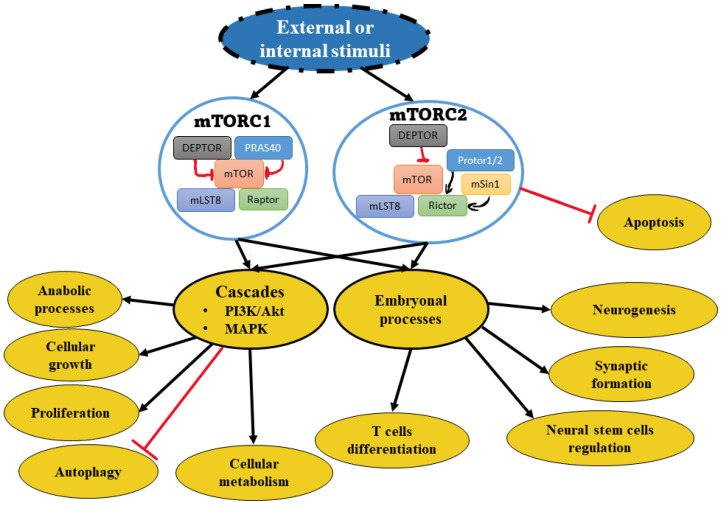
Schematic view of mTOR complexes and relevant pathways. The depiction shows mTOR complexes that induce downstream pathway activation. The activation involves either or both cascade cellular activities and/or embryonal processes under development.

Dysregulation of the more complexes (mTORC1 and mTORC2) are involved in many pathophysiological processes, including neurodegeneration, metabolic disorders, and tumorigenesis (Figure 2) [16]. The hyperactivation of mTOR underpins the mechanisms of multiple cancers. This overactivation is mainly secondary to the genetic mutations, activation of upstream molecules, or loss of inhibitory proteins such as PTEN [12,16]. This hyperactivation suggests mTOR resistance to treatment.

### 3.2. mTOR Involvement in Medulloblastoma

In medulloblastoma, mTOR has a fundamental role in tumor development through many pathways. The Sonic hedgehog (SHH) pathway is critical for cell fate determination and embryonic neuronal development, and it is implicated in multiple cancers, including the SHH subgroup in medulloblastoma. SHH is a secreted protein which when bound to a cellular membrane protein PTCH1, releases its inhibitor on the smoothed homolog (Smo). The Smo protein transduces Gli protein which is a transcription factor, leading to downstream gene expression adjustments [17]. mTORC1 affects this process through a translational effect on Smo, resulting in the hyperactivation and proliferation of granule neuron precursor cells [18]. The mTOR pathway is implicit in the development of medulloblastoma stem cells (cancer stem cells), resulting in treatment failure [6]. It is known that cancer stem cells are resistant to conventional therapy [19]. The crosstalk between mTOR and Hedgehog (HH) pathways has been reported to stimulate medulloblastoma progression. This crosstalk promotes mRNA translation by stimulating the expression of eukaryotic translation initiation factor 4E (eIF4E) and inhibiting eukaryotic translation initiation factor 4E-binding protein 1 (4EBP1), leading to medulloblastoma tumor progression [18]. The factor eIF4E is present downstream of PI3K. In inhibition of both proteins may induce synergistic effect as reported in breast cancer cell lines [20]. Similarly, dual targeting of HH and PI3K/mTOR pathways in a medulloblastoma mouse model, inhibited tumor growth and extended survival of tumor-bearing animals [21]. In addition, SHH-driven medulloblastoma is inhibited by targeting the mTOR pathway [18]. Taken together, these findings suggest that dual targeting mTOR signaling pathway in addition to other pathways is a promising therapeutic approach to overcome resistance in treating medulloblastoma patients.

Most of the medulloblastoma molecular subgroups harbor genetic and epigenetic alterations that activate the PI3K/Akt/mTOR pathway to fuel cancer progression [22,23]. For instance, various genetic alterations in the PIK3CA gene have been detected in both group 4 and the WNT subgroup. The PI3K/Akt/mTOR pathway activation has also been reported within the MYC-driven group 3 medulloblastoma [24]. Interestingly, the combination of PIK inhibitors with histone deacetylase inhibitors (HDACI), reduced the tumor growth of MCY-driven medulloblastoma in vivo, providing a novel therapeutic approach to treating the most aggressive form of medulloblastoma. Recently, a combination of siRNA and small molecule inhibitors to target the MYC and mTOR pathways in MYC-driven medulloblastoma cells resulted in reducing cell growth and survival in vitro and prolonged survival of MYC-driven medulloblastoma xenografts [25]. In addition to the contribution of genetic and epigenetic activation of The PI3K/Akt/mTOR pathway in medulloblastoma progression, aberrant constitutive activation of several growth factor receptors acting upstream of the PI3K/Akt/mTOR pathway has also been linked with the development and progression of some of the medulloblastoma subgroups. For instance, medulloblastoma group 4, which is considered the largest and the most diverse group, harbors multiple molecular alterations that constitutively activate several growth factor receptors, including the insulin-like growth factor (IGF), platelet-derived growth factor (PDGF), epidermal endothelial growth factor (EGFR) and vascular endothelial growth factor (VEGF) among others [26,27,28]. It has been suggested that targeting cell membrane receptors that are highly expressed on medulloblastoma cells could be one of the effective therapeutic approaches to medulloblastoma treatment. In that direction, Snuderl et.al, reported that placental growth factor (P1GF) and its receptor neuropilin1 (Nrp1) are highly expressed on the majority of medulloblastoma cells and their expressions are associated with poor survival and metastasis in patients [29] Blocking P1GF/Nrp1 in medulloblastoma animal models resulted in disease regression, reduction of metastasis and prolonged survival of treated animals [29]. All these findings suggest that the mTOR signaling is a key regulator of medulloblastoma development and progression and is considered a therapeutic target for treating medulloblastoma patients in combination with other current therapies.

### 3.3. mTOR-Targeted Therapy in Medulloblastoma

**Figure 3:**
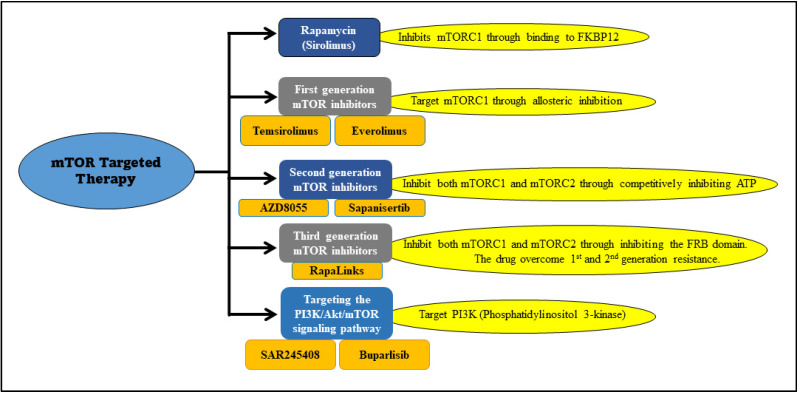
mTOR inhibitors and their targets. The depiction illustrates specific inhibitors and their targets used for mTOR-targeted therapy.

Multiple drugs have been reported to target mTOR through different binding sites or mechanisms. Rapamycin inhibits mTORC1 through binding to FK506 Binding Protein 12 (FKBP12). This forms a complex which binds to FKB12-Rapamycin Binding (FRB), an active site at mTOR. The complex triggers a structural modification that causes the fragmentation of Raptor and mTOR binding [30]. Similarly, rapamycin analogs or first-generation mTOR inhibitors such as temsirolimus and everolimus, are targeting mTORC1 and causing disintegration through allosteric inhibition with altered pharmaceutical properties (Figure 3) [4,31].

Temsirolimus is a prodrug of sirolimus [32]. Sirolimus (rapamycin) was initially approved for immunosuppression, followed by studies investigating other potential roles, including its antineoplastic effect. Sirolimus has completed a phase I clinical trial for pediatric solid tumors ((NCT01331135) such as ewing sarcoma, osteosarcoma, glioblastoma multiforme, ependymoma, and medulloblastoma [33]. The study goals were to examine the tolerated doses, the safety profile of sirolimus, understand the correlation with the mTOR effector pS6 kinase, and the risk of infections. In total, 18 patients were treated with escalating doses of sirolimus in combination with celecoxib, etoposide, and cyclophosphamide. Only two medulloblastoma patients were included in this study. The study found that the drugs were well tolerated, with a decrease in the lymphocyte count CD4 as a side effect, and the pS6 levels were undetectable in all the sirolimus dosing regimens [33]. 

Temsirolimus (CCI-779) is formed through the esterification of rapamycin, making it more water-soluble and easily administered through the intravenous route. It is converted to rapamycin through carboxylesterases, specifically by CYP3A4 and CYP3A5 [32]. Temsirolimus is a kinase inhibitor which has been studied in multiple cancers such as renal cancer, ovarian cancer, lymphoma and neuroblastoma [4]. It completed a phase III clinical trial for refractory renal cell carcinoma with significant increase in survival rate compared to the interferon-alpha version of the drug. It has been approved by FDA for renal cell carcinoma [34]. Temsirolimus has been evaluated in medulloblastoma within three phases I clinical trials which were evaluating multiple pediatric solid tumors. The first clinical trial included 18 patients of refractory solid tumors who were treated with temsirolimus, and it was well tolerated, except for nausea as the major dose-limiting toxicity [35]. In this trial, only two medulloblastoma patients were recruited. Similarly, to everolimus, the phosphorylations of AKT, pS6, and 4EBP1 were decreased; however, it was not related to dose or clinical response [35]. The second study enrolled 71 pediatric patients with relapsed tumors. This study has only two medulloblastoma patients’ recruited. The study participants were treated with an increasing dose of intravenous (IV) temsirolimus, oral irinotecan and temozolomide. These treatments were well tolerated, except for hyperlipidemia as dose-limiting toxicity and few adverse events [36]. The third phase I clinical trial was to investigate temsirolimus combined with perifosine in pediatric solid tumors (Table 1) [37]. Perifosine is an antitumor alkyl phospholipid (APL) that targets AKT [38]. The study goals were to determine the toxicity, the dosing amount, the pharmacokinetics of the medications, and the effects of the combination of the AKT inhibitor with the mTOR inhibitor. In total, 22 patients were recruited for the combination therapy, which was safe and well-tolerated. There was a linear correlation between the perifosine dose and response. However, significant plasma level variability was documented in patients which prevents proper assessment of side effects [37]. A preclinical xenograft study was conducted using a medulloblastoma model implanted PNET/MB cell line (DAOY) that is rapamycin-sensitive. The model was treated with rapamycin, temsirolimus, and cisplatin, which had an additive cytotoxicity effect. The single-agent treatment with temsirolimus decreased growth significantly after one week of treatment [39].

Everolimus is an analog with higher bioavailability than sirolimus [40]. It has been investigated in clinical trials for various tumors, such as non-small cell carcinoma, mantle cell lymphoma, Rhabdomyosarcoma Sarcoma, and medulloblastoma (NCT00187174) [40]. Everolimus is FDA approved for neuroendocrine tumors of pancreatic origin (PNET) [40]. A clinical trial phase I to assess everolimus was reported for medulloblastoma and other pediatric tumors. The maximum tolerated dose of everolimus was evaluated through 41 participants. The study demonstrated inhibition of the mTOR pathway in peripheral blood mononuclear cell including downstream molecules p70-S6 kinase, and AKT. These molecules usually get activated by mTORC2 stimulation cascade [41]. The treatment was well tolerated in children with solid tumors. Minimal pS6 kinase activity and a decrease in AKT phosphorylation were observed after therapy. However, in the adult population increase in phosphorylation and higher expression of mTORC2 were observed post-treatment. A possible theory for the treatment failure was the development of resistance against first-generation mTOR inhibitor [41,42].

The second-generation mTOR inhibitors (Figure 3) affect both mTORC1 and mTORC2 through competitively binding to the ATP sites. They also have a PI3K inhibitory effect [4]. Many inhibitors are being tested for various cancers such as GSK2126458 and gedatolisib, but not yet tested on medulloblastoma [26]. AZD8055 is a potent second-generation inhibitor with mTORC1, mTORC2, and PI3K inhibitory effects (Table 1) [43]. It completed a phase I clinical trial in patients with solid tumors, including breast, lung, and pancreatic cancers, but is still in a preclinical (animal studies) phase regarding medulloblastoma [44]. There is a study used medulloblastoma xenograft (BT-50) cells with AZD8055 treatment resulted in a stable disease status [45]. Similarly, another drug called sapanisertib (MLN0128) was tested on medulloblastoma (BT-28 cells) xenograft, which induced disease stabilization but not regression [46]. 

The third-generation mTOR inhibitors, also called RapaLinks, were developed to overcome resistant mutations of the first and second generations in the FKBP12-rapamycin binding (FRB) or kinase domain mutants through an avidity-based approach [47]. This generation does not inhibit rapamycin binding to FKBP12 or the FRB domain of mTOR. It delivers MLN0128 to inhibit the ATP site of the mTORC1 complex. The third generation showed promising results after they induced initial regression in glioblastoma [48]. No clinical studies have been conducted on the third generation against medulloblastoma malignancy. 

Alternative inhibitors have targeted other components of the PI3K/Akt/mTOR signaling pathway in medulloblastoma. Some of these components are PI3K and Akt. Different medulloblastoma cell lines were treated with buparlisib (BKM120), an oral PI3K inhibitor that exhibited significant growth inhibition through stimulating apoptosis caspases and downregulating mTOR and AKT downstream molecules [49]. A preclinical study that used a different PI3K inhibitor, called pilaralisib (SAR245408), had a similar conclusion [50]. In addition, the Sonic hedgehog (SHH) pathway, which is intertwined with the PI3K/Akt/mTOR signaling pathway, has been involved in medulloblastoma-targeted therapy [5,16]. Some of these medications were FDA-approved for basal cell cancer, such as vismodegib (GDC-0449 and Sonidegib (NVP-LDE225) [51]. Both medications inhibit the target pathway through binding to Smo. Vismodegib underwent a phase II clinical trial for SHH-activated medulloblastoma; however, the study was terminated as the number of successful cases was not achieved (Table 1) [52].

### 3.4. mTOR Treatment Resistance in Medulloblastoma

The development of chemotherapeutic agents for medulloblastoma faces many challenges, such as crossing the blood-brain barrier with varying compositions of the different types. Treatment resistance remains the most challenging aspect of medulloblastoma therapy. One of the challenges is represented in the existence of cancer stem cells [6]. Those cells generate a variety of heterogeneous offspring. Similarly, Dysregulation of microRNA expression has implicated another challenge to therapy. Various contributing pathways have been established. For instance, Notch, Sonic hedgehog, WNT, and mTOR/AKT/PIK3 pathways have been reported in medulloblastoma stem cell preservation, a crucial reason for relapse and treatment failure [53]. In addition, Smo inhibition in an SHH-activated medulloblastoma cell line became resistant through the activation of the RAS/MAPK or AKT/PIK3 pathways [54,55]. Gedatolisib inhibited medulloblastoma tumor growth in a preclinical study which also reported the treatment ability to cross the blood-brain barrier. The drawback was in the treatment’s ability to highly bind to animals and human plasma proteins. This suggests the requirement of higher doses to achieve the therapeutic target. Development of resistance was reported in PTCH+/− medulloblastoma model cells which were treated with Sonidegib. When the PI3K inhibitor, NVP-BKM120, was added to the treatment, the resistance rate was significantly delayed. It is worth noting that PI3K signaling promotes Smo and its downstream effector Gli2 [56]. Smo possesses another route of resistance in medulloblastoma that could be overcome through the inhibition of p90 ribosomal S6 kinase (RSK), which plays a functional role in pediatric medulloblastoma and is part of the MAPK pathway. Additionally, medulloblastoma-resistant cells to Smo inhibitors were increasingly sensitized by silencing RSK1/2 [57]. 

The combination of PI3K inhibitors and mTORC1 inhibitors reduces the resistance of the over-activated PI3K pathway. Another combination therapy used alpelisib (PI3Kα inhibitor) and OSI-027 (mTOR inhibitor) through augmenting PI3K and mTOR inhibitors’ antineoplastic effect. This was demonstrated on DAOY and group 3 medulloblastoma *in vitro* cell line models. The study described pronounced apoptosis rate in the DAOY cell line, most likely due to treatment inhibitory effect on PI3K activity and in the SHH-derived cancer. Interestingly, inhibition of PI3KA and mTOR suppressed the self-renewal ability of the cancer stem cells and neurosphere formation significantly more than the PI3KA knockdown or mTOR inhibition alone. The study highly recommended PI3K and mTOR dual targeting, especially in Smo inhibitor-resistant SHH-derived medulloblastoma cells [58]. 

With mTOR resistance being an emerging topic in medicine, few studies investigated the kinase resistance mechanisms in medulloblastoma. The two studies included in our analysis targeted this topic. The first study investigated a mechanism of mTOR treatment failure in immunosurveillance tumor escape mechanisms through IDO1 involvement. IDO1 is a regulator of inflammation, especially T cell-mediated immunity [59]. It induces the amplification of regulatory T cells (Treg), preventing an appropriate immunological response against tumor cells [60]. In the study, Folgiero et al. reported mTOR and IDO1 expression in all subgroups of medulloblastoma human tissue specimens [61]. In a separate experiment with the DAOY cell line, the addition of the mTOR inhibitor rapamycin induced the expression of IDO1 and increased the tumor immune tolerance. This suggests a crosstalk mechanism between the two molecules, which could be a contributing factor to the resistance of mTOR treatment. The study found this effect only in medulloblastoma and not in other brain tumors, such as ganglioglioma and glioblastoma. They advocated against the use of mTOR inhibitors in medulloblastoma as a single agent [61]. It is worth noting that a regulatory interaction was documented between Notch and mTOR, involving T cell activities [62]. These activities need more attention from the scientific community.

The second study by Eckerdt et al. found another mechanism of resistance through an alternative pathway activation through the Mnk2-eIF4E loop [63]. The eukaryotic translation initiation factor (eIF4E) is one of the effector mediators of mTOR [6]. Its activation leads to cell proliferation and survival. Another mechanism of its activation following the mTOR inhibitor was with MAP (mitogen-activated protein) kinase-interacting kinases (Mnk), specifically Mnk2-mediated phosphorylation in rapamycin-treated DAOY cells, indicating a Mnk2-eIF4E feedback loop. Although treatment with OSI-027, a second-generation mTOR inhibitor, did not induce eIF4E phosphorylation, the effect was independent of mTORC2. In the study, two cell lines have been used. The DAOY cells in this study most likely represent the SHH subgroup as it was positive for PTCH1 and SHH, and the CD556 line cells were positive for MYCC amplification, similar to group 3 medulloblastoma. When both cell lines (DOAY and CD556) were treated with CGP57380, an inhibitor of Mnk, the antitumor effect of the mTOR inhibitors was maximized, suggesting a possible therapeutic advantage of combined Mnk2 and mTOR inhibitors [63].

### 3.5. mTOR Treatment Resistance in Other Cancers

Several mechanisms have a potential role in drug resistance, such as genetic mutations, Compensatory pathways activation, epigenetic transformations, and metabolic alterations have been identified [64]. Those mechanisms are partially investigated in medulloblastoma and need to be promoted. Genetic point mutations were observed in many cancers. For example, mutations in FPR1 (FKBP12), TOR2, and TOR1 impair the mTOR inhibitors and FKBP12 interaction [65]. The S2035F mutation in the FRB domain of mTOR was found in a rapamycin-resistant breast cancer BT474 cell line, which interfered with the mTOR–FKBP12 interaction [66]. Similarly, in another breast cancer cell line, MCF, which has a mutation at the M2327I position of the kinase domain, rendered these cells resistant to AZD8055 [47]. Compensatory pathways were observed secondary to mTORC1 or mTORC2 inhibition through the involvement of insulin receptors and insulin-like growth factors (IGF) [64]. The mTORC1 and its effector p70S6K1 downregulate insulin and IGF through the inhibition of insulin receptor substrate (IRS). The IRS is responsible for the PI3K/AKT pathway activation and PIP3 synthesis, which stimulates anabolic cellular growth [64]. The reason for colon cancer cell resistance to everolimus was theorized to be alteration in MEK/ERK signaling pathways and its overexpression in many neoplastic processes [67]. Similarly, another resistance mechanism shows high WNT/β-catenin activity in colorectal cancer (CRC), which is highly associated with mutations in the tumor suppressor APC with mTOR signaling [64,68]. This was demonstrated in a cell line of CRC resistant to gedatolisib, a PI3K and mTOR inhibitor. The resistance most likely resulted from a frameshift mutation of the T cell factor, which positively regulates WNT/β-catenin and is associated with high glycogen synthase kinase (GSK3β) expression, a molecule that controls WNT/β-catenin both positively and negatively. Inhibition of GSK3β rendered resistant cells more sensitive to mTOR inhibition [69]. When glioblastoma (GBM) cells were treated with PI3K and mTOR inhibitors, MSK1 was upregulated, phosphorylates β-catenin and increases its activity [70]. A different mechanism of mTOR inhibition resistance in glioblastoma is GSK3β-dependent, without the WNT/β-catenin effect, but through its microtubule-associated protein (MAP)1B [64,71]. Interestingly, increased glutamine catabolism resulting in increased oncogenesis was found after mTOR inhibitor administration., However, glutaminase inhibition resulted in substantial growth restriction in glioblastoma [72]. Another example of metabolic alteration is through the stimulation of the purine salvage pathway since PI3K/mTOR-resistant small-cell lung carcinoma cells have higher AMP, GMP, and hypoxanthine levels [73]. Since some of the mTOR resistant pathways are shared between medulloblastoma and other cancers, the resistant mechanisms could also appear in medulloblastoma and promote treatment.

### 3.6. The Advancement of mTOR Treatment 

The use of drugs from other medical disciplines, such as immunosuppression, is a traditional way to discover new therapies. However, the success in other organs does not mean success in medulloblastoma. The route is tedious and sometimes rewarding. It is an excellent way to reduce patients suffering time, especially if the treatment is FDA approved. Although validation is required, fewer procedures will be performed. More preclinical studies are required to discover treatments that will resolve mTOR resistance. It is known that cancer stem cells are contributing to medulloblastoma progression. It is also understood that cancer stem cells initiate the recurrence of the disease. Cancer stem cell-specific drugs are limited, though they would contribute significantly to lower toxicity or side effects. The molecular findings of all four subgroups of medulloblastoma are involved in the activities of the various types of stem cells. The same genes may act as factors to maintain the stem cells and self-renewal process. WHO 2021 guide has kept the four medulloblastoma subgroups as the most common ones. However, it acknowledges the identification of 13 subgroups for the disease. Moreover, separating and diagnosing those subgroups will need the involvement of scientific research [74].

Personalized medicine is playing a great role in treating cancer, especially medulloblastoma. Molecular testing is already in serving personalized medicine. The use of next-generation sequencing in personalized medicine is debatable. The vast quantity of data produced per patient may have raised a barrier against accurate treatment. This initiates a debate that more information is not always beneficial, but rather confusing. The availability of information without knowing whether it is positive or negative will add pressure on physicians who need to review all the data before assigning a treatment plan. This challenge stimulated the identification of disease signatures through next-generation sequencing. However, the field of medulloblastoma is still progressing in terms of this route [75]. 

Gene therapies are also competitive and opened a new door for the successful treatment of medulloblastoma. The inhibition of mTORC1 or mTORC2, but not both, has been a challenge to biochemists when representing the third generation of mTOR inhibitors. This process may need the attention of molecular geneticists to provide a platform for CNS (Central Nervous System) cancers, including medulloblastoma. The target is not limited to the mTOR complexes but includes downstream pathways. Fighting cancer is only one aspect of gene therapy. In addition, some therapeutic side effects are permanent, stimulating the need to use gene therapy to resolve some of the symptoms. Improving the quality of life through gene therapy is a legitimate goal for research. An additional aspect of gene therapy is to improve existing treatment. Occasionally, it is better to use gene therapy combined with other treatments, rather than a sole treatment. The ability of gene therapy to stimulate or inhibit genes may focus on overriding drug resistance, side effects, or even the prognosis when used with treatment combinations.

The ultimate goal is to cure cancer. We suggest simultaneously investigating both the second-and third-generation anti-mTOR inhibitors against medulloblastoma. The rationale of using different generations is to override drug resistance. The aggressive treatment could potentially benefit patients by using multiple drug generations at the same time since they have different binding sites. However, this approach may increase toxicity and side effects.

This study provides a comprehensive explanation and systematic literature search regarding the mechanistic resistance of the mTOR inhibitor. Since the first- and second-generation drugs of the mTOR inhibitors developed strong resistance in medulloblastoma, two preclinical studies identified the routes of resistance. The two identified studies related to the mechanism of resistance of mTOR inhibitors involve IDO1 and Mnk2 and eIF4E genes [61,63]. These genes contribute to treatment failure. The suggested routes open a new path for failed drugs to succeed by targeting the source of resistance in synergy with approved inhibitors. Unfortunately, most of the first- and second-generation mTOR inhibitors used in clinical trials did not evaluate enough cases of medulloblastoma (Table 1). This suggests a lack of clinical knowledge in terms of the management of resistance of the medulloblastoma mTOR inhibitor. The treatment description of mTOR inhibitor resistance has no clinical guideline, mandating more clinical research.

## 4. Limitations

This review was limited to English-language articles listed in PubMed or Google Scholar. The two articles found are preclinical in vitro studies, with no in vivo or animal model studies. Both mainly focused on the first-generation mTOR inhibitor resistance, and there was no investigation of the effect of conventional chemotherapy combinations. In the first study of IDO1 involvement in the mTOR pathway, there is no clear mechanism of IDO1 induction, and no specific genetic subgroup was correlated with the resistance, which could impede the clinical use. Similarly, in the second study investigating the Mnk2-eIF4E feedback loop formation secondary to mTORC1 inhibition, a precise mechanism of Mnk2 induction is required.

## 5. Conclusions

The mTOR pathway plays one of the most fundamental roles in cancer development. It is intertwined with various pathways, and it is pivotal to understand the inhibition resistance, especially in highly resistant tumors, such as medulloblastoma. The two studies demonstrated the possible resistance mechanisms in medulloblastoma, including immune evasion and alternative phosphorylation by Mnk2. The literature is deficient in mTOR involvement in medulloblastoma and mTOR inhibitor resistance, with mTOR having potential as a chemotherapeutical agent for medulloblastoma. Similarly, the availability of treatment against cancer stem cells requires serious attention, as a minimum effort was exerted to identify third-generation therapies against medulloblastoma. 

## Figures and Tables

**Table 1:** mTOR inhibitors clinical trials in medulloblastoma (MD). The table summarizes completed and current clinical trials that used mTOR inhibitors in the treatment of medulloblastoma.

Drug	Target	Patients Groups	Medulloblastoma Cases/Total Tumor Cases	Phase	Status/ Result	The National Clinical Trial Number
Sirolimus in combination with metronomic therapy	mTOR	Children with recurrent or refractory solid and brain tumors	2 / 18	I	Complete/ well tolerated	NCT01331135
Everolimus	mTOR	Pediatric patients with refractory solid tumors	3 / 41	I	Complete/ well tolerated	NCT00187174
Temsirolimus	mTOR	Pediatric patients with recurrent/refractory solid tumors	2 / 71	I	Complete/ did not meet efficacy	NCT00106353.
Temsirolimus in combination with irinotecan and temozolomide	mTOR	Children, adolescents, and young adults with relapsed or refractory solid tumors	2 / 72	I	Complete/ tolerated dose	NCT01141244
Temsirolimus with perifosine	mTOR AKT	Recurrent pediatric solid tumors	2 / 23	I	Complete/ tolerable toxicity	NCT01049841
Vismodegib in combination with temozolomide versus temozolomide alone	Smo mTOR	Patients with medulloblastomas with an activation of the Sonic hedgehog pathway	24 / 24	I II	Terminated/ unclear	NCT01601184

## Data Availability

All data available within the text.
